# Case report and literature analysis on individualized hysteroscopic management of complex Müllerian anomalies complicated by bilateral adenomyosis

**DOI:** 10.3389/fmed.2026.1804421

**Published:** 2026-07-09

**Authors:** Hui Liao, Rena, Shu-han Wang, Niang-hai Peng, Yan Ke, Xiao-lei Song

**Affiliations:** 1 Guangzhou University of Chinese Medicine, Guangzhou, China; 2 Shenzhen Clinical College of Integrated Chinese and Western Medicine, Shenzhen, China

**Keywords:** adenomyosis, hysteroscopy, levonorgestrel-releasing intrauterine system, suture fixation technique, uterus didelphys with single vagina

## Abstract

**Objective:**

To investigate the clinical characteristics and individualized hysteroscopic conservative treatment strategies for a case of uterine didelphys with a single vagina complicated by bilateral adenomyosis, adenomyoma, and endometrial polyps.

**Methods:**

A retrospective analysis was conducted on the clinical data of a reproductive-aged female patient admitted to Shenzhen Hospital of Integrated Traditional Chinese and Western Medicine, Guangzhou University of Chinese Medicine. The patient’s medical history, imaging features, surgical approach, and follow-up outcomes were systematically reviewed and analyzed in conjunction with relevant literature.

**Results:**

The patient presented with progressively worsening dysmenorrhea and menorrhagia. Imaging revealed uterine didelphys (single vaginal type) with bilateral diffuse adenomyosis and adenomyoma, accompanied by endometrial polyps. Hysteroscopic polypectomy was performed. Based on the morphology of both uterine cavities and the characteristics of the lesions, a levonorgestrel-releasing intrauterine system was suture-fixed hysteroscopically in the right uterine cavity, while a GyneFix^®^ IUD was placed in the left cavity. At the 12-month follow-up, the patient’s dysmenorrhea VAS score decreased from 9 to 2, and the pictorial blood loss assessment chart (PBAC) score decreased from 280 to 35, with no device expulsion or lesion recurrence observed.

**Conclusion:**

Hysteroscopic individualized selection and suture fixation of intrauterine devices can serve as an effective uterus-preserving treatment option for patients with complex uterine anomalies complicated by adenomyosis.

## 1 Introduction

Adenomyosis (AM) is a common chronic benign gynecological disorder among women of reproductive age, characterized pathologically by the invasion of endometrial glands and stroma into the myometrium. Its clinical manifestations predominantly include progressive dysmenorrhea, menorrhagia, and abnormal uterine bleeding (1). Imaging studies indicate that diffuse adenomyosis is the most prevalent subtype, typically presenting on magnetic resonance imaging (MRI) as diffuse thickening of the junctional zone (JZ) (> 12 mm), ill-defined borders, and predominantly low signal intensity on T2-weighted imaging (T2WI), often accompanied by adenomyomatous nodules. These features are key contributors to the refractory nature of symptoms and poor response to medical therapy (2, 3).

Müllerian duct anomalies (MDAs), resulting from abnormal development of the paramesonephric ducts during the embryonic period, occur in approximately 4–7% of the general female population (4). Among these, uterus didelphys with a single vagina (UD) represents a relatively rare subtype (AFS class III), characterized by complete separation of bilateral uterine bodies and cervices with a shared single vaginal structure. Previous studies indicate that the co-occurrence of UD with adenomyosis is uncommon, affecting only about 2–5% of cases, with involvement typically limited to one uterine side (5).

When congenital reproductive tract anomalies coexist with bilateral diffuse adenomyosis, adenomyomas, and endometrial polyps, the superimposition of uterine anatomical abnormalities and lesion burden substantially increases diagnostic and therapeutic complexity. Current literature predominantly reports hysterectomy as the definitive treatment, with exceedingly scarce documentation on uterus-preserving individualized hysteroscopic interventions for bilateral uterine involvement (6–8).

This article presents a case of a reproductive-aged female with uterus didelphys and a single vagina, complicated by bilateral diffuse adenomyosis, adenomyomas, and endometrial polyps. After suboptimal responses to multiple conservative treatments, an innovative individualized hysteroscopic strategy was implemented following comprehensive imaging evaluation. The procedure included hysteroscopic polypectomy and differential intrauterine device placement in each uterine cavity. Notably, a levonorgestrel-releasing intrauterine system (LNG-IUS) was sutured and secured under direct hysteroscopic guidance in one cavity. This report summarizes the diagnostic and therapeutic approach along with follow-up outcomes, providing a reference for conservative management of similarly complex cases.

### 1.1 General information

The patient is a 38-year-old married female, gravida 1 para 1 (G1P1), who was admitted on June 24, 2024, due to “progressive worsening dysmenorrhea accompanied by increased menstrual flow for 4 years.” Her menstrual cycle is 30–35 days, with a duration of 7 days and heavy flow accompanied by blood clots. The visual analog scale (VAS) score for dysmenorrhea was 8–9. She previously underwent high-intensity focused ultrasound (HIFU) and pharmacological therapy for uterine fibroids at another institution in 2020, with suboptimal therapeutic outcomes. In the past year, she has experienced brownish discharge preceding menstruation and prolonged menstrual duration. Past medical history includes surgery for pulmonary minimally invasive carcinoma and multiple prior gynecological surgeries. She has a history of one full-term spontaneous delivery. Gynecological examination upon admission revealed a palpable double uterus, both in anteverted position, enlarged to the size of a 2-month pregnancy, firm in consistency, mobile, and non-tender. The patient currently has one child and expresses a strong desire to preserve the uterus, wishing to maintain reproductive function while undergoing treatment.

Admission diagnoses:

① Adenomyosis uteri

② Abnormal uterine bleeding

③ Uterine malformation: Uterus didelphys with single vagina

### 1.2 Imaging characteristics

**FIGURE 1:**
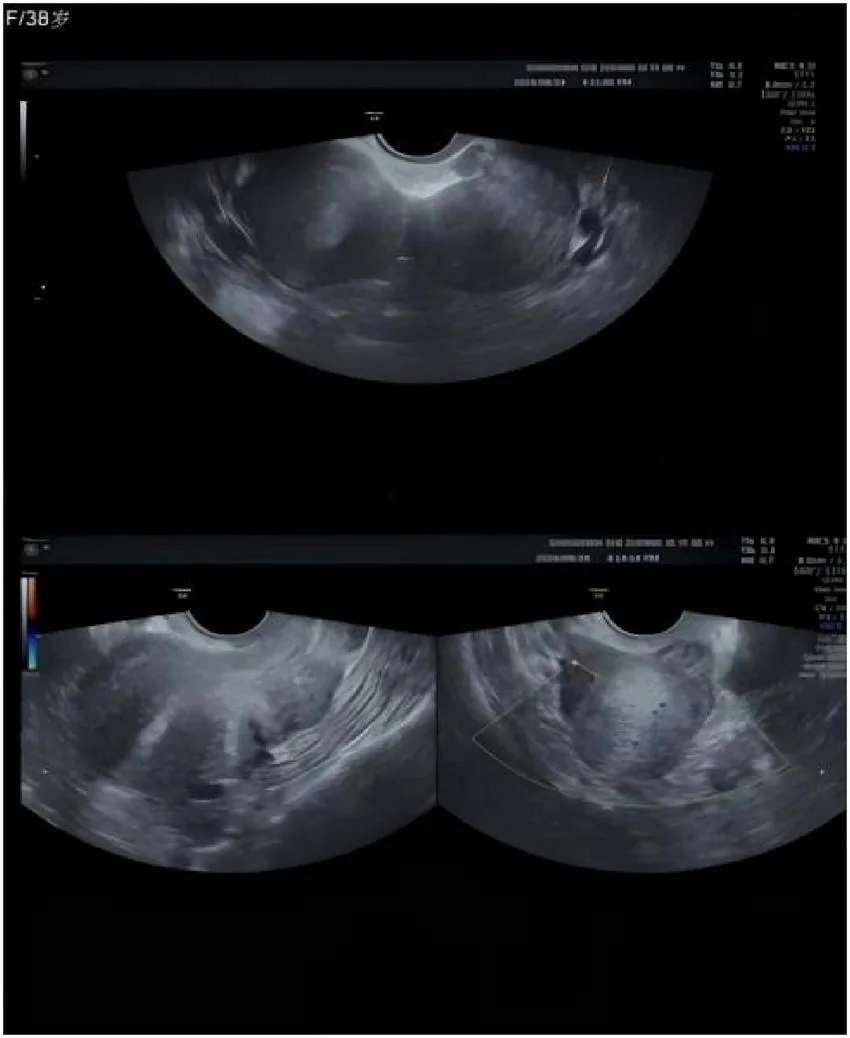
Preoperative transvaginal three-dimensional ultrasound showing uterus didelphys (single-vagina type) complicated by bilateral diffuse adenomyosis.

Pelvic MRI performed on March 29, 2024, at Union Shenzhen Hospital, Huazhong University of Science and Technology revealed: Uterus didelphys (single vagina type) and bilateral uterine fibroids (largest measuring approximately 16 × 12 mm). Transvaginal three-dimensional ultrasound (TVS-3D) conducted after admission on June 24, 2024, demonstrated: clearly visualized bicornuate uterine structure, heterogeneous myometrial echogenicity with multiple nodular changes bilaterally, the largest measuring approximately 32 × 24 × 31 mm (posterior wall of the right uterus). The junctional zone appeared thickened with ill-defined borders in some regions. Color Doppler flow imaging (CDFI) showed scattered punctate blood flow signals. Endometrial thickness was approximately 7–8 mm bilaterally. Imaging diagnosis: Uterus didelphys complicated by diffuse adenomyosis with adenomyomas (Figure 1).

### 1.3 Pathological findings

**FIGURE 2:**
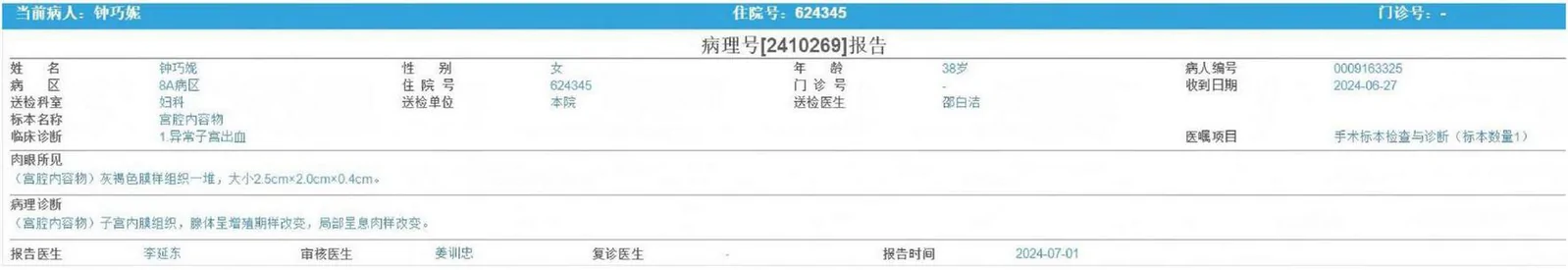
Postoperative histopathological report showing endometrial tissue with proliferative-phase changes and focal polypoid changes; no malignancy identified.

Histopathological analysis of the uterine cavity contents revealed endometrial alterations consistent with a proliferative phase pattern, with focal polypoid architectural features. No evidence of malignancy was identified (Figure 2).

### 1.4 Operative procedure

After providing fully informed consent and discussing alternative approaches including hysterectomy, bilateral placement of non-fixed LNG-IUS, and conservative medical management, the patient elected to undergo hysteroscopic surgery under general anesthesia. Intravenous cefuroxime was administered 30 min preoperatively for infection prophylaxis. Cervical dilation was performed progressively using Hegar dilators from smaller to larger sizes up to No. 10, with uterine sounding measuring a depth of 10 cm.

The procedure involved hysteroscopic examination and resection of bilateral endometrial polyps. An individualized intrauterine device placement strategy was formulated based on the distinct anatomical structures and pathological characteristics observed in each uterine cavity (Figures 3–10).

**FIGURE 3:**
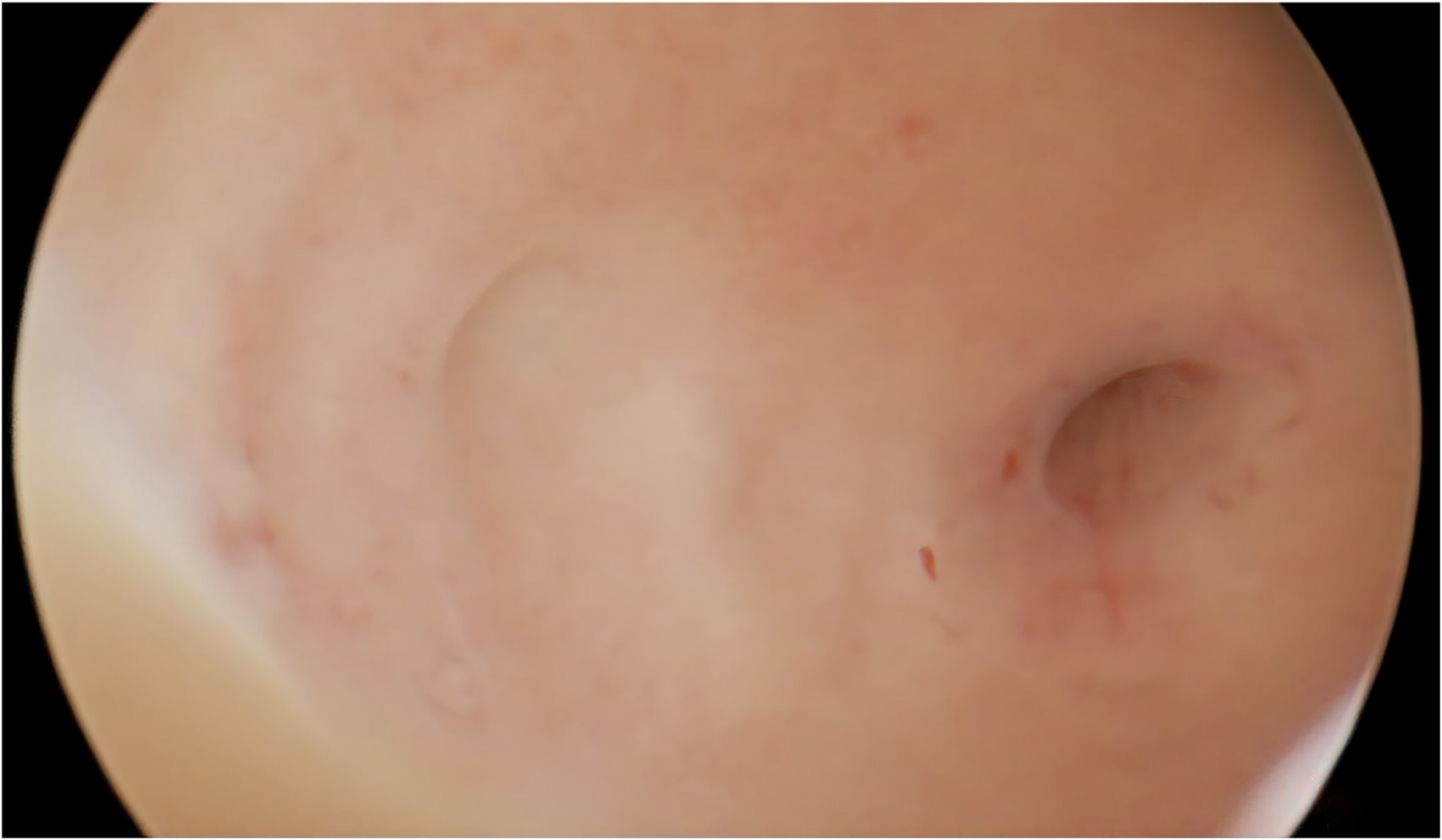
Hysteroscopic views of LNG-IUS (Mirena) placement and suture fixation in the right uterine cavity.

**FIGURE 4:**
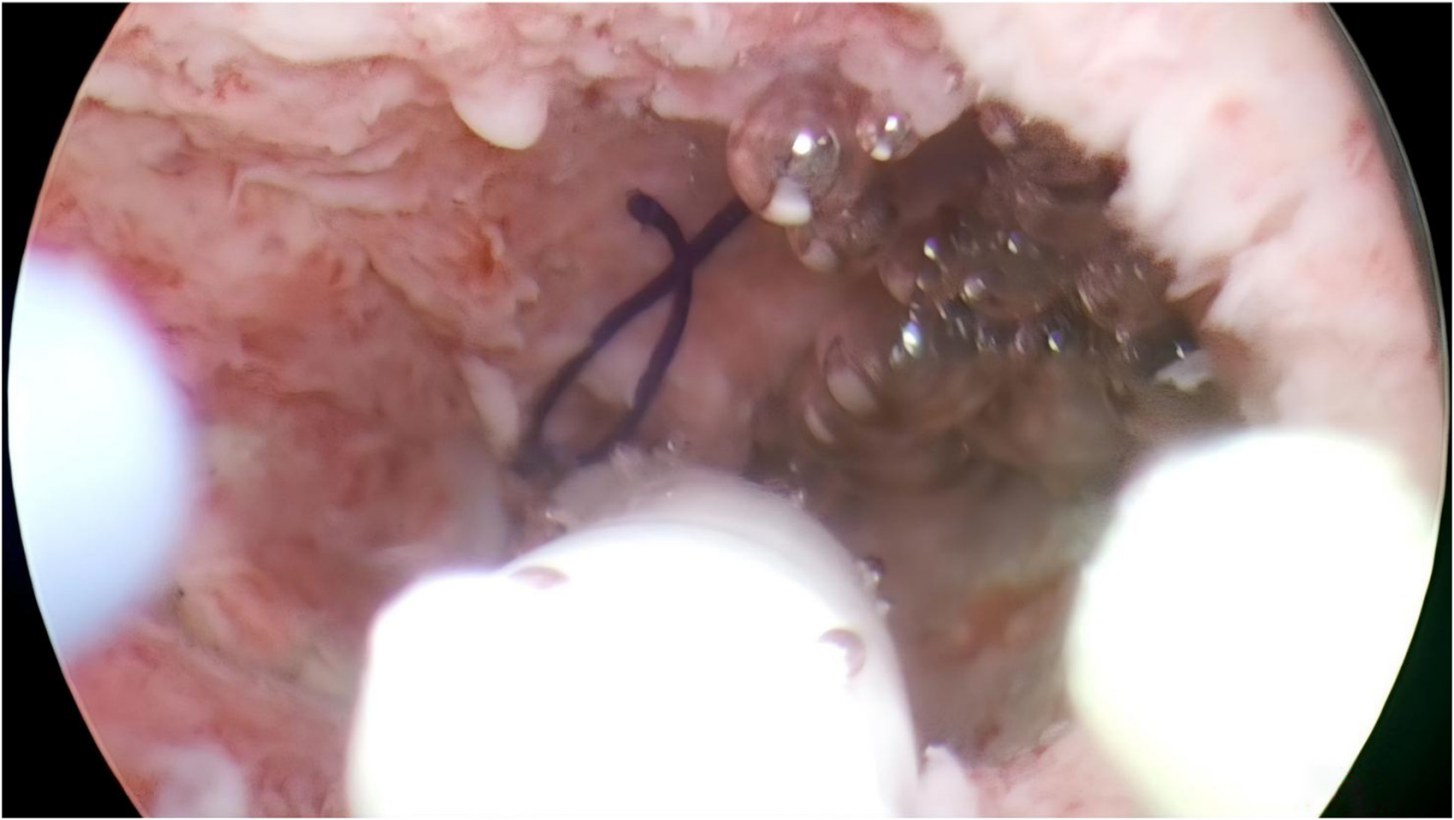
Hysteroscopic views of GyneFix^®^ copper IUD insertion and anchoring in the left uterine cavity.

**FIGURE 5:**
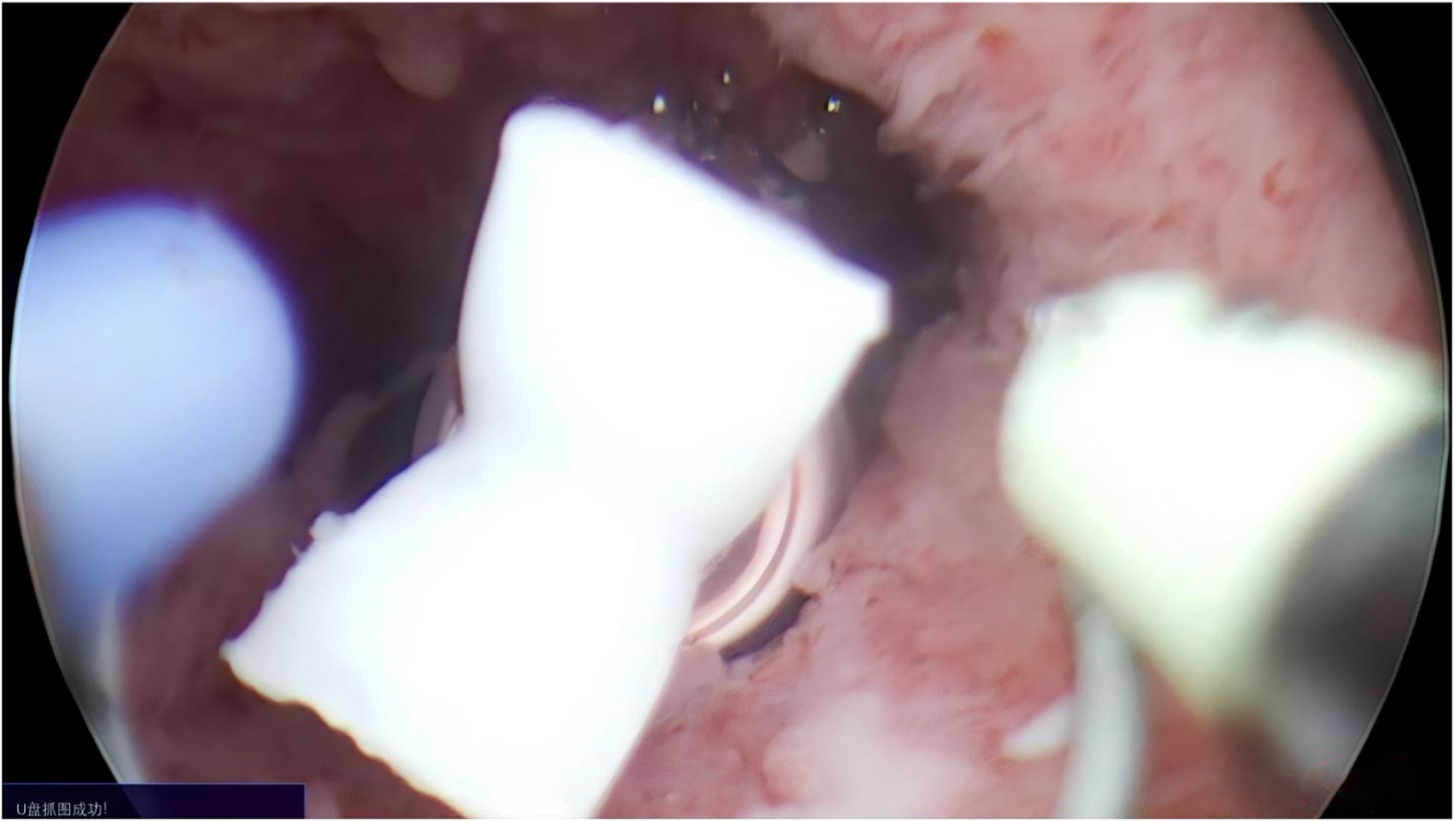
Postoperative trend curves showing VAS scores for dysmenorrhea and PBAC scores for menstrual blood loss from preoperatively to 12 months postoperatively.

**FIGURE 6:**
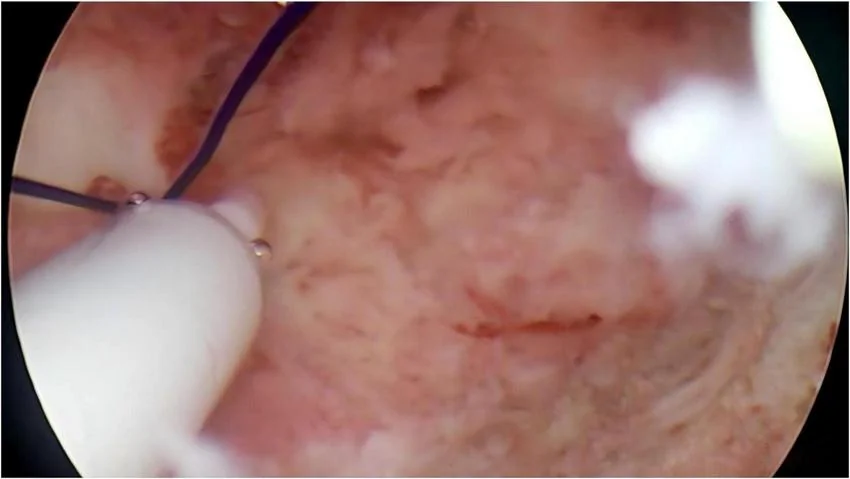
Preoperative hysteroscopic view of the left uterine cavity before IUD placement.

**FIGURE 7:**
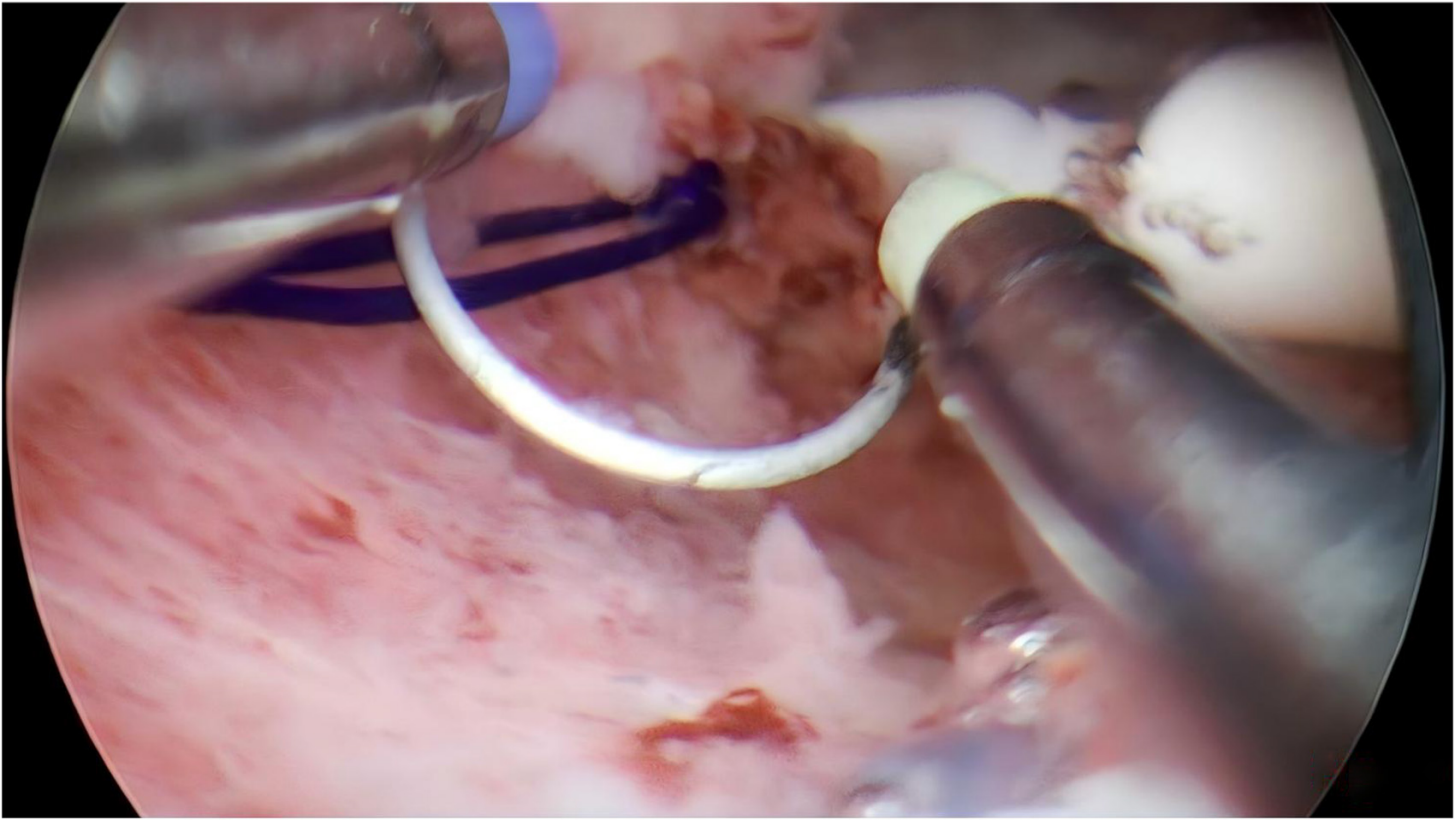
Intraoperative hysteroscopic view during GyneFix^®^ IUD deployment in the left uterine cavity.

**FIGURE 8:**
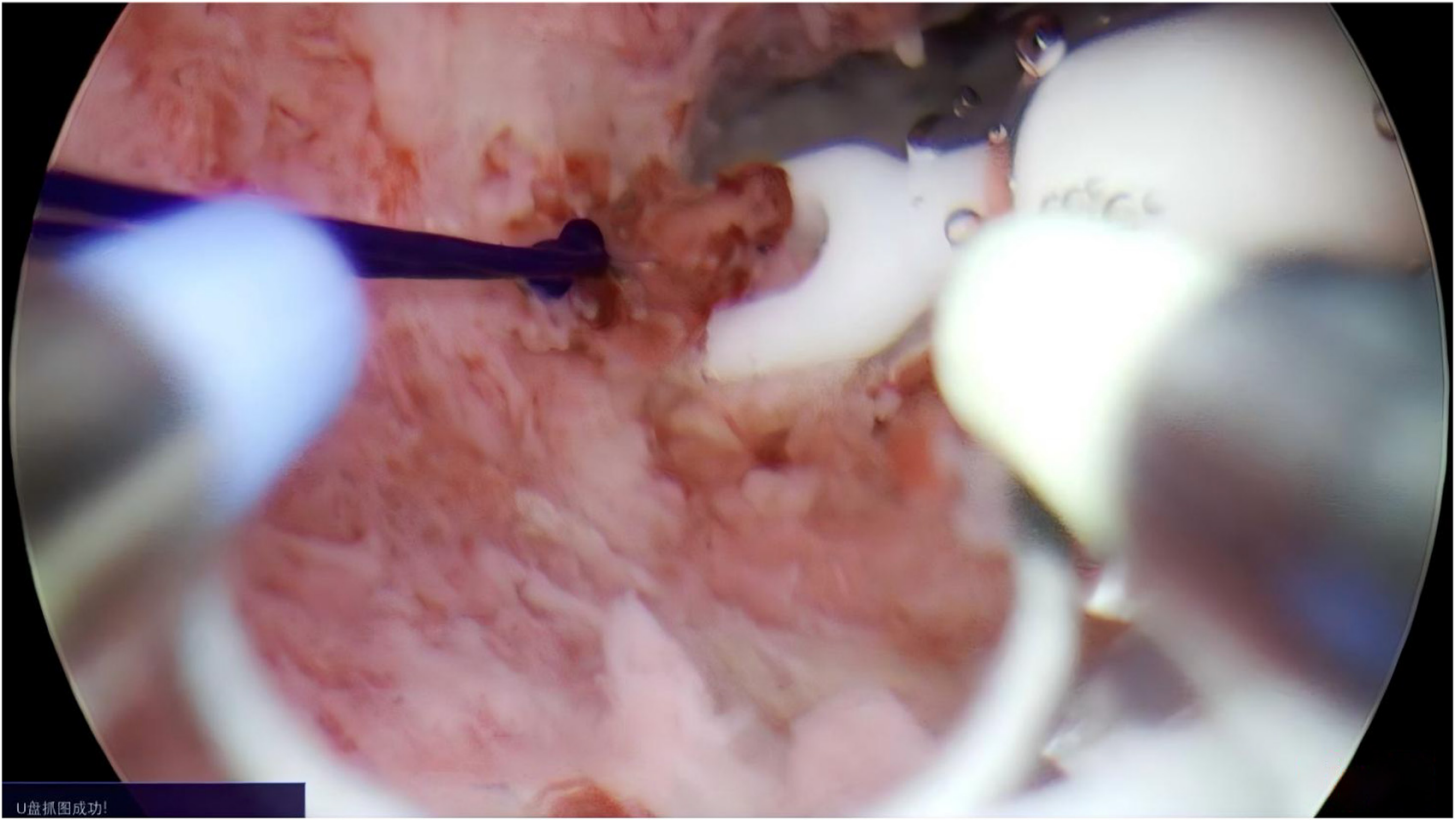
Hysteroscopic confirmation of the sutured LNG-IUS position in the right uterine cavity.

**FIGURE 9:**
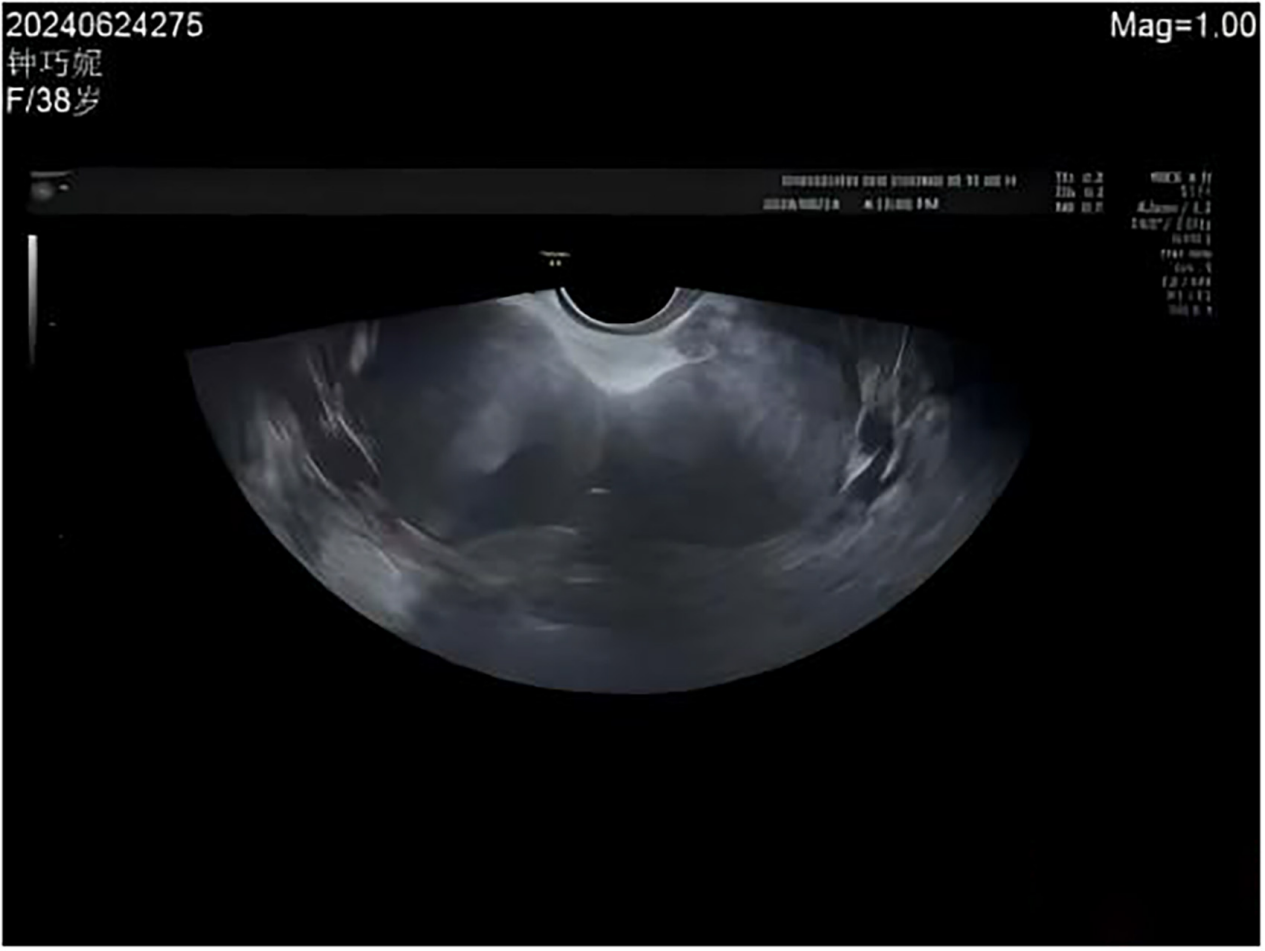
Hysteroscopic confirmation of the anchored GyneFix^®^ IUD position in the left uterine cavity.

**FIGURE 10:**
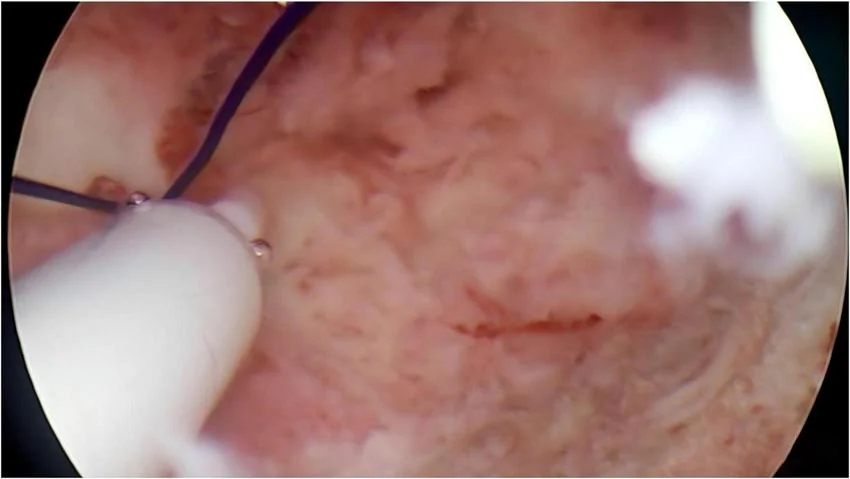
Close-up view of the suture fixation knot for the LNG-IUS at the uterine fundus.

Intrauterine Device Placement on the Right Side: Given the capacious right uterine cavity and the diffuse distribution of adenomyotic lesions, a levonorgestrel-releasing intrauterine system (LNG-IUS, brand name: Mirena^®^) was selected to achieve efficient and sustained local therapy. To ensure long-term stability and prevent displacement or expulsion within the enlarged uterine cavity, the Mirena device was sutured to the myometrium of the right uterine wall under hysteroscopic guidance using a 2–0 non-absorbable polypropylene suture. Due to the observed friability of the endometrial tissue, two continuous sutures were applied to enhance fixation strength by increasing the suturing width. The suture was secured with a surgeon’s knot. Repeat hysteroscopy confirmed proper positioning of the device, and traction testing demonstrated firm anchorage (Supplementary Video 1).

Left Uterine Cavity Procedure: The left uterine cavity exhibits a relatively elongated morphology, which provides optimal anatomical suitability for the placement of a frameless fixed intrauterine device. After comprehensive evaluation of anatomical compatibility, cost-effectiveness, and patient tolerance, the GyneFix^®^ intrauterine system was selected for insertion. Under hysteroscopic visualization, the anchoring node of the GyneFix device was securely implanted into the myometrium at the uterine fundus, with endoscopic confirmation of ideal positioning (Supplementary Video 2).

**FIGURE 11:**
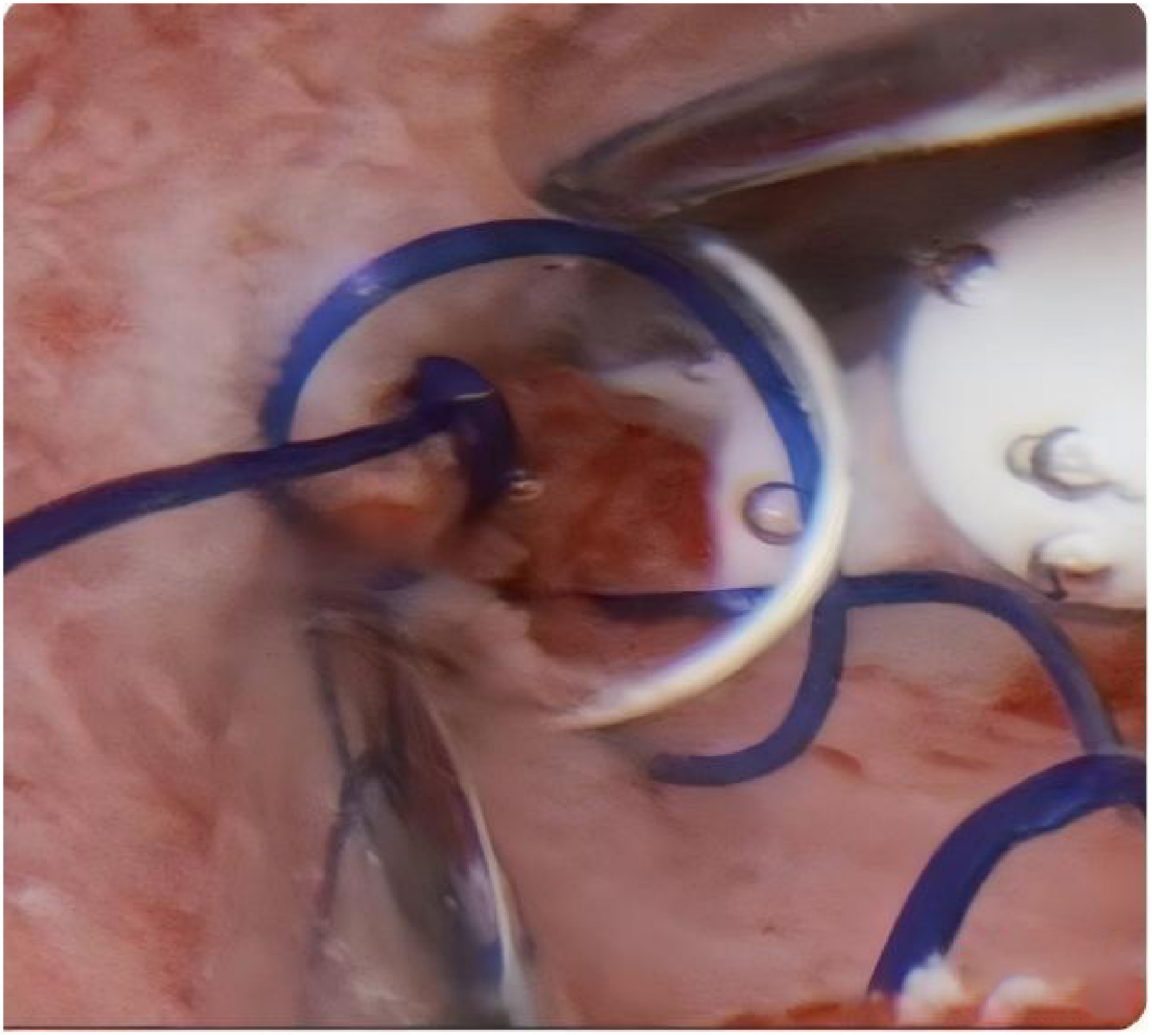
Intraoperative hysteroscopic view of the fundal suture anchoring technique for LNG-IUS, showing the secure myometrial anchor point.

**FIGURE 12:**
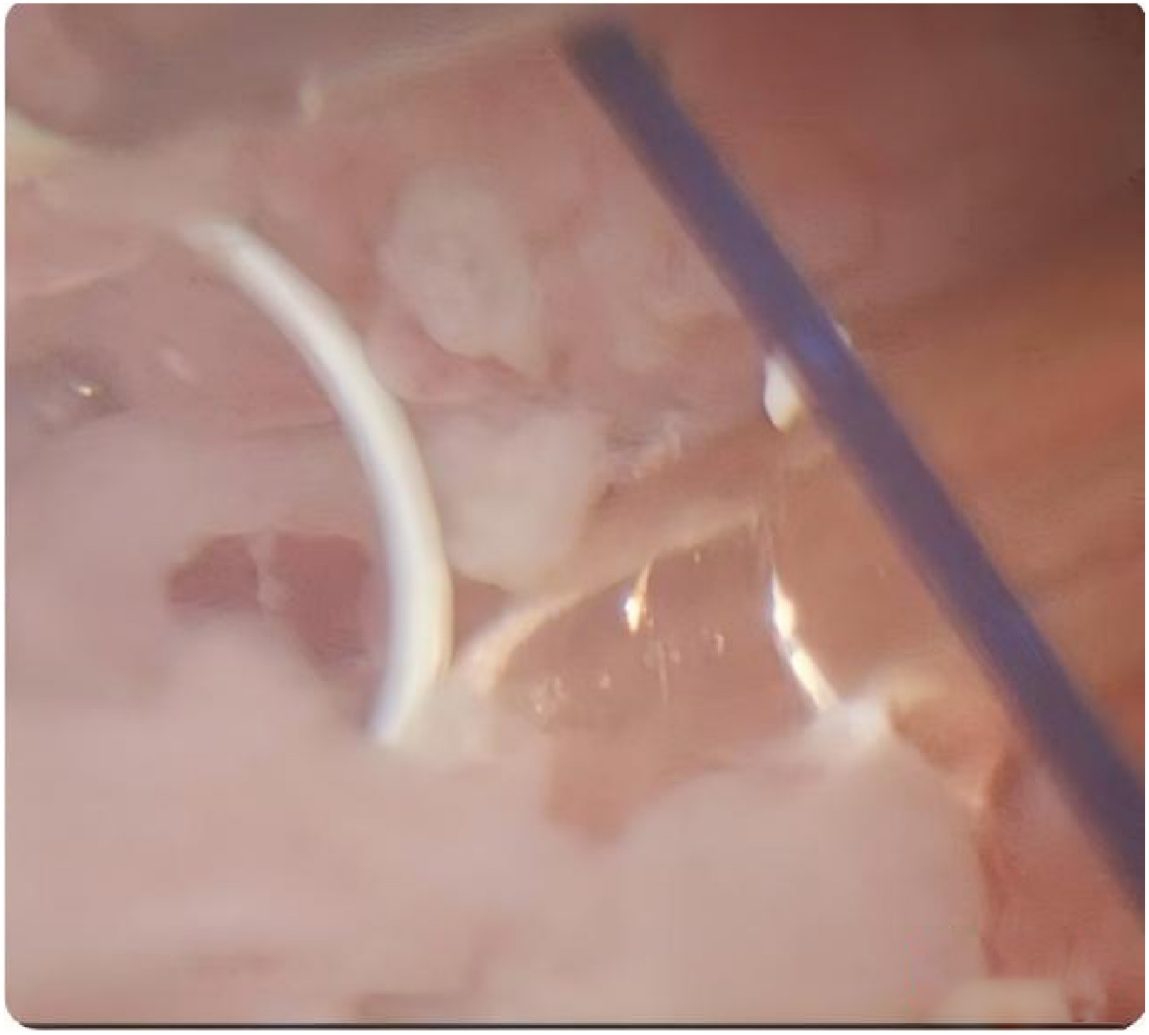
12-month follow-up transvaginal ultrasound confirming stable LNG-IUS position in the right uterine cavity, with no signs of migration or adenomyosis progression.

The procedure was completed successfully, with a total operative duration of 85 min (Figure 11). Manually fixed and sutured Mirena^®^ IUD in the right uterine cavity (Figure 12) GyneFix^®^ IUD placed in the left uterine cavity.

### 1.5 Follow-up results

The 12-month postoperative follow-up revealed a marked alleviation of the patient’s dysmenorrhea symptoms, with the Visual Analog Scale (VAS) score decreasing from 9 points preoperatively to 2 points. Menstrual volume was significantly reduced, as evidenced by the Pictorial Blood Loss Assessment Chart (PBAC) score dropping from 280 points to 35 points. Imaging follow-up demonstrated no recurrence of adenomyosis lesions. Both intrauterine devices were properly positioned without evidence of displacement or expulsion.

At the 12-month postoperative follow-up, significant alleviation of dysmenorrhea symptoms was observed, with the Visual Analog Scale (VAS) score decreasing from 9 to 2. Menstrual blood loss was markedly reduced, as indicated by the Pictorial Blood Loss Assessment Chart (PBAC) score dropping from 280 to 35. Imaging reevaluation revealed no recurrence of adenomyosis lesions. Both intrauterine devices were properly positioned without evidence of displacement or expulsion (Supplementary Video 3 shows the postoperative hysteroscopic confirmation of device positioning).

## 2 Discussion

### 2.1 Diagnostic and therapeutic challenges of uterus didelphys with bilateral adenomyosis and consideration of alternative approaches

Adenomyosis co-occurring with uterus didelphys is inherently uncommon, and the presentation of diffuse adenomyosis with adenomyomas bilaterally is exceptionally rare. This structural characteristic limits the efficacy of conventional pharmacotherapy or standalone interventional treatments, while significantly elevating the risk of LNG-IUS displacement or expulsion. Previous literature predominantly documents unilateral uterine involvement in cases of uterus didelphys with adenomyosis, with hysterectomy frequently serving as the definitive treatment. In the present case, both uteri exhibited symmetrical diffuse adenomyotic changes, accompanied by adenomyomas and endometrial polyps, which substantially complicated conservative management. During therapeutic decision-making, we thoroughly evaluated and communicated multiple alternatives to the patient: while total hysterectomy would achieve disease eradication, the patient strongly desired uterine preservation and declined this option; medical therapy alone is non-invasive, yet the patient had a history of suboptimal response to both HIFU and prior medications, indicating limited expected efficacy; conventional placement of an LNG-IUS (without fixation) in the right uterine cavity posed a high risk of expulsion. Literature reports indicate that the 3-year cumulative expulsion rate of LNG-IUS in adenomyosis patients can reach 11.1%, markedly higher than the 4.6% observed in women with normal uteri (9). Moreover, among patients with a history of IUD expulsion, the rate of re-expulsion within 1 year may be as high as 31.4% (10). Thus, the central challenge in this case was achieving long-term stable therapeutic outcomes while preserving uterine integrity and reproductive potential.

### 2.2 Rationale for hysteroscopic suture fixation of LNG-IUS in adenomyosis

The levonorgestrel-releasing intrauterine system (LNG-IUS) has demonstrated efficacy in alleviating dysmenorrhea and abnormal uterine bleeding in patients with adenomyosis. However, risks of device displacement and expulsion are significantly elevated in cases involving uterine enlargement, abnormal uterine cavity morphology, cervical incompetence, or concurrent uterine anomalies (e.g., didelphic uterus) (10). The present case—characterized by severe pathology in the right uterine cavity with irregular morphology—represents a typical high-risk scenario for expulsion. Consequently, hysteroscopically guided suture fixation of the LNG-IUS was performed to enhance device stability and prolong therapeutic efficacy. This technique has been validated to significantly improve device retention rates, and follow-up outcomes in this case confirm its effectiveness in controlling dysmenorrhea and menstrual blood loss. A recent review by Zhao et al. (11) indicated that the expulsion rate of LNG-IUS in adenomyosis patients ranges from 15 to 37.5% (compared to only 3%–10% in normal uteri), with key risk factors including uterine enlargement (> 150 mL), intrauterine lesions, and insertion technique. Preventive strategies such as GnRH-a pretreatment and image-guided placement show promise in high-risk populations. These findings align closely with the proactive approach of suture fixation adopted in this case to mitigate expulsion risk.

### 2.3 Value of a personalized “differential bicavitary” strategy

It is noteworthy that this case deviated from the conventional approach of placing an LNG-IUS in both uterine cavities. Instead, a differential selection of intrauterine devices was made based on the anatomical and pathological differences between the two cavities. This decision was guided by the principle of “precision therapy and individualized placement”: the right cavity exhibited extensive lesions and severe pathology, necessitating an efficient and sustained drug delivery system to manage the advanced adenomyosis—hence the choice of an LNG-IUS (Mirena^®^) combined with an innovative hysteroscopic suturing fixation technique to mitigate expulsion risk. In contrast, the left cavity’s morphology was more compatible with the frameless design of the GyneFix^®^ IUD, which adapts better to abnormal uterine shapes, reduces foreign body sensation and rejection reactions, and entails relatively lower costs. Placement of the GyneFix ring in the left cavity not only accommodated the irregular anatomy effectively but also avoided potential adverse effects, such as persistent spotting associated with bilateral high-dose progestin release. This combined regimen effectively controlled symptoms while taking into account patient tolerance and financial burden, reflecting a highly individualized hysteroscopic treatment philosophy.

**FIGURE 13:**
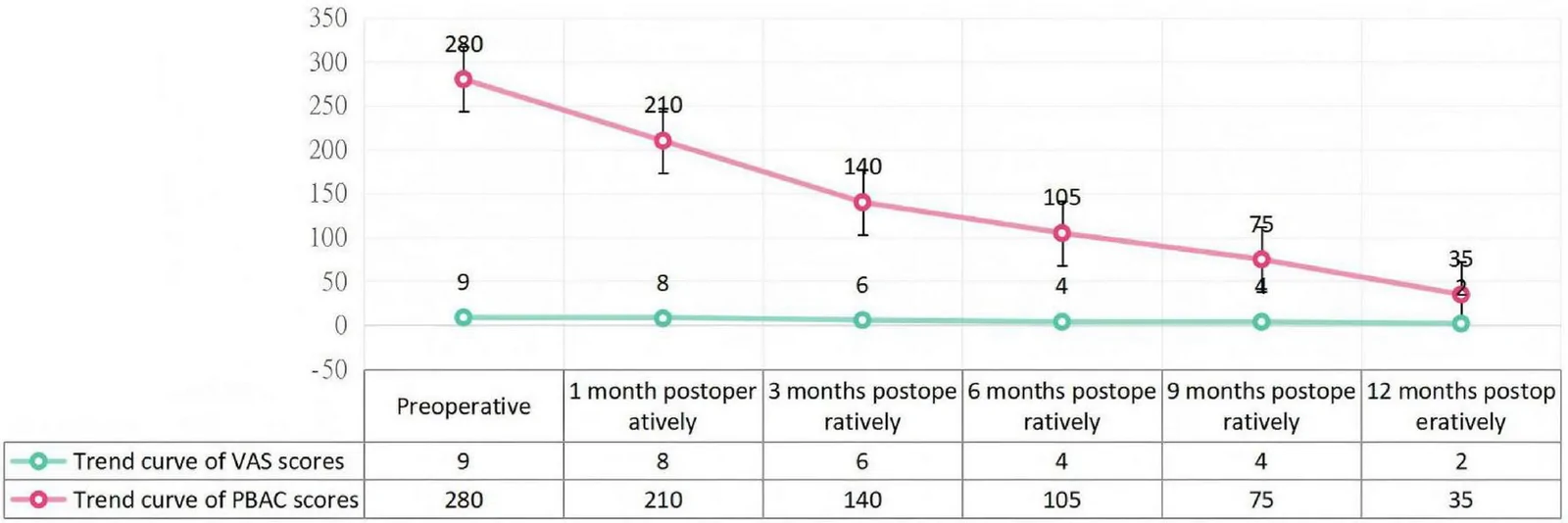
Final hysteroscopic confirmation of the GyneFix^®^ IUD position in the left uterine cavity, verifying secure anchoring.

In terms of mechanism of action, although two different types of intrauterine devices (IUDs) were placed bilaterally in the uterine cavities, their core mechanisms for symptom alleviation may be similar and complementary. The levonorgestrel-releasing intrauterine system (LNG-IUS) achieves endometrial atrophy through the continuous local release of levonorgestrel, resulting in reduced menstrual bleeding. Concurrently, it downregulates estrogen receptors in the endometrium, suppresses endometrial proliferation, and thereby alleviates dysmenorrhea. Although the GyneFix-IUD is an inert scaffold, its mechanism of fixation to the uterine fundus ensures device stability. It induces a local aseptic inflammatory response that alters the endometrial environment, thereby preventing abnormal bleeding or pain caused by displacement. The combined action of both devices comprehensively reduces overall menstrual volume and pain intensity. Notably, the localized pharmacological effect of the LNG-IUS may cover both uterine cavities simultaneously, providing a plausible mechanism for bilateral symptomatic improvement, which warrants further investigation. At the 12-month postoperative follow-up, significant symptomatic improvement was observed: the dysmenorrhea Visual Analog Scale (VAS) score decreased from 9 to 2, and the Pictorial Blood Loss Assessment Chart (PBAC) score dropped from 280 to 35. Transvaginal three-dimensional ultrasound confirmed normal positioning of both IUDs without displacement or expulsion. No adverse events such as infection, embedment, or perforation occurred during the treatment period. This study has certain limitations, as some imaging findings were not further analyzed and require exploration in subsequent research. This therapeutic approach not only effectively controlled symptoms but also preserved the patient’s fertility, offering a novel strategy for managing such complex cases of uterine malformation accompanied by adenomyosis (Figure 13).

## 3 Conclusion

For this patient with complex reproductive tract malformations combined with bilateral diffuse adenomyosis, based on precise assessment by three-dimensional ultrasound and MRI, we attempted an individualized hysteroscopic treatment strategy: after removing bilateral endometrial polyps, we placed different intrauterine devices according to the anatomical features and lesion severity of both sides of the uterine cavity. For the right uterine cavity with a higher risk of device detachment, we applied hysteroscopic suture fixation of LNG-IUS. The 12-month follow-up showed significant improvement in dysmenorrhea and menstrual volume, and imaging confirmed stable device positions on both sides and no progression of lesions. This experience suggests that this individualized minimally invasive strategy has initial feasibility in controlling symptoms, avoiding device detachment risks, and preserving the anatomical structure of the uterus. It is important to note that this study is only a single case report with a limited follow-up period, and the above findings cannot be regarded as evidence of efficacy. Further validation is needed through studies with larger sample sizes and longer follow-up periods. This case provides an exploratory and yet-to-be-further-evaluated clinical approach for conservative treatment of complex uterine malformations combined with severe adenomyosis.

## Data Availability

The original contributions presented in this study are included in the article/Supplementary material, further inquiries can be directed to the corresponding author.

## Supplementary material

Supplementary Video 1 End of right uterine cavity procedure.

Supplementary Video 2 End of left uterine cavity procedure.

Supplementary Video 3 Postoperative follow-up confirmation.

## References

[1] Zhang H Cao B Tong J Guo J Zheng J Zhu L et al. An innovative surgical approach: suture fixation of the levonorgestrel-releasing intrauterine system in the treatment of adenomyosis. *BMC Womens Health*. (2022) 22:451. 10.1186/s12905-022-01932-6 36384588 PMC9670435

[2] Dason ES Maxim M Sanders A Papillon-Smith J Ng D Chan C et al. Guideline No. 437: diagnosis and management of adenomyosis. *J Obstet Gynaecol Can.* (2023) 45:417–429.e1. 10.1016/j.jogc.2023.04.008. 37244746

[3] Chinese Medical Association Gynecologists Branch, Chinese Society of Obstetrics and Gynecology Endometriosis Collaborative Group. Guidelines for diagnosis and treatment of endometriosis (3rd edition). *Chin J Obstet Gynecol.* (2021) 56:812–24. 10.3760/cma.j.cn112141-20211018-00603 34954958

[4] Grimbizis GF Camus M Tarlatzis BC Bontis JN Devroey P. Clinical implications of uterine malformations and hysteroscopic treatment results. *Hum Reprod Update*. (2001) 7:161–74. 10.1093/humupd/7.2.161 11284660

[5] Saravelos SH Cocksedge KA Li TC. Prevalence and diagnosis of congenital uterine anomalies in women with reproductive failure: a critical appraisal. *Hum Reprod Update*. (2008) 14:1665–75. 10.1093/humupd/dmn018 18539641

[6] Chan SP Yuen PM Cheung LP Chung TK Lam CW Haines CJ et al. The prevalence of congenital uterine anomalies in infertile women with recurrent miscarriage: a case-control study. *Hum Reprod.* (2011) 26:2723–30. 10.1093/humrep/der228

[7] Zhang T He SS Yang YG. Congenital uterus didelphys complicated with endometriosis: a case report and literature review. *China Med Pharm.* (2024) 14:191–4.

[8] Sun FQ Duan H Gan L Wang S Sun XW Liu Y et al. Uterus didelphys with bilateral adenomyosis and left uterine leiomyoma: a case report and literature review. *Chin J Family Plann Obstet Gynecol.* (2016) 8:71–4.

[9] Youm J Lee HJ Kim SK Kim H Jee BC. Factors affecting the spontaneous expulsion of the levonorgestrel-releasing intrauterine system. *Int J Gynaecol Obstet*. (2014) 126:24825498. 10.1016/j.ijgo.2014.02.017 24825498

[10] Deng T Zhou YF. Risk factors and preventive strategies for malposition and expulsion of the levonorgestrel-releasing intrauterine system. *Chin J Obstetrics Gynecol.* (2019) 54:780–3.

[11] Zhao J Chen J Wang X Wang H Li CQ Wei B et al. A narrative review and risk-stratification framework for preventing LNG-IUS expulsion in adenomyosis. *Ther Clin Risk Manag.* (2026) 22:566786. 10.2147/TCRM.S566786 41859203 PMC12998664

